# Diagnosis and Management of Gastrointestinal Manifestations in Children with Cystic Fibrosis

**DOI:** 10.3390/diagnostics14020228

**Published:** 2024-01-22

**Authors:** Dana-Teodora Anton-Păduraru, Alina Mariela Murgu, Laura Iulia Bozomitu, Dana Elena Mîndru, Codruța Olimpiada Iliescu Halițchi, Felicia Trofin, Carmen Iulia Ciongradi, Ioan Sârbu, Irina Mihaela Eṣanu, Alice Nicoleta Azoicăi

**Affiliations:** 1 Department of Mother and Child Medicine, “Grigore T. Popa” University of Medicine and Pharmacy, 700115 Iaṣi, Romania; dana.anton@umfiasi.ro (D.-T.A.-P.); laura.bozomitu@umfiasi.ro (L.I.B.); mindru.dana@umfiasi.ro (D.E.M.); olimpiada.iliescu@umfiasi.ro (C.O.I.H.); alice.azoicai@umfiasi.ro (A.N.A.); 2 “Sf. Maria” Children Emergency Hospital, 700309 Iasi, Romania; carmen.ciongradi@umfiasi.ro (C.I.C.); sarbu.ioan@umfiasi.ro (I.S.); 3 Department of Preventive Medicine and Interdisciplinarity–Microbiology, “Grigore T. Popa” University of Medicine and Pharmacy, 700115 Iaṣi, Romania; felicia.trofin@umfiasi.ro; 4 2nd Department of Surgery, Pediatric Surgery and Orthopedics, “Grigore T. Popa” University of Medicine and Pharmacy, 700115 Iaṣi, Romania; 5 Medical Department, “Grigore T. Popa” University of Medicine and Pharmacy, 700115 Iaṣi, Romania; irina.esanu@umfiasi.ro

**Keywords:** cystic fibrosis, children, gastrointestinal manifestations, diagnosis, management

## Abstract

Cystic fibrosis (CF) is primarily known for its pulmonary consequences, which are extensively explored in the existing literature. However, it is noteworthy that individuals with CF commonly display gastrointestinal (G-I) manifestations due to the substantial presence of the cystic fibrosis transmembrane conductance regulator (CFTR) protein in the intestinal tract. Recognized as pivotal nonpulmonary aspects of CF, G-I manifestations exhibit a diverse spectrum. Identifying and effectively managing these manifestations are crucial for sustaining health and influencing the overall quality of life for CF patients. This review aims to synthesize existing knowledge, providing a comprehensive overview of the G-I manifestations associated with CF. Each specific G-I manifestation, along with the diagnostic methodologies and therapeutic approaches, is delineated, encompassing the impact of innovative treatments targeting the fundamental effects of CF on the G-I tract. The findings underscore the imperative for prompt diagnosis and meticulous management of G-I manifestations, necessitating a multidisciplinary team approach for optimal care and enhancement of the quality of life for affected individuals. In conclusion, the authors emphasize the urgency for further clinical studies to establish a more robust evidence base for managing G-I symptoms within the context of this chronic disease. Such endeavors are deemed essential for advancing understanding and refining the clinical care of CF patients with G-I manifestations.

## 1. Introduction

Cystic fibrosis (CF) is an intricate multiorgan disorder affecting epithelial organs, including the respiratory tract, exocrine pancreas, intestine, hepatobiliary system, sweat glands, and, more recently, myeloid cells in secretory vesicle membranes [1,2,3]. It stands as the most prevalent autosomal recessive monogenic disease, exhibiting a chronic, progressive, and potentially fatal course in the Caucasian population. The condition involves a generalized dysfunction of the exocrine glands, particularly those producing mucus, resulting in the clinical triad of exocrine pancreatic insufficiency, chronic lung disease, and elevated chloride and sodium concentrations in sweat [4]. The gene responsible for encoding the cystic fibrosis transmembrane conductance regulator (CFTR) protein, a 1480-amino acid protein, was identified in 1989, with over 2000 mutations discovered to date and F508del representing 75% of mutations in Europe and North America [2].

Six pathogenic molecular mechanisms elucidate the extent of CFTR impairment, including protein-production defects (class I), processing defects (class II), regulatory defects (class III), conduction defects (class IV), reduced synthesis (class V), and decreased CFTR stability or impaired regulation of other channels (class VI) [2,4,5].

CF is predominantly renowned for its extensively studied pulmonary manifestations, yet it is noteworthy that these patients frequently experience gastrointestinal (G-I) problems due to the robust expression of the CFTR protein throughout the intestine [6,7]. The spectrum of G-I manifestations is diverse, encompassing exocrine pancreas involvement, meconium ileus, distal intestinal obstruction syndrome (DIOS), constipation, small intestinal bacterial overgrowth (SIBO), and intestinal inflammation. These manifestations significantly impact the quality of life and long-term prognosis [8], establishing them as the foremost nonpulmonary aspects of CF. Consequently, recognizing and effectively managing G-I manifestations hold paramount importance for maintaining health and enhancing the quality of life in CF patients.

The primary objectives of this study were to synthesize knowledge on G-I manifestations of CF, offering comprehensive insights into each G-I manifestation, the diagnostic methods, and the potential advancements in therapeutic strategies. This includes novel treatments addressing the fundamental effects of CF on the G-I tract and their outcomes.

## 2. Material and Methods

### 2.1. Search Strategy

**Figure 1:**
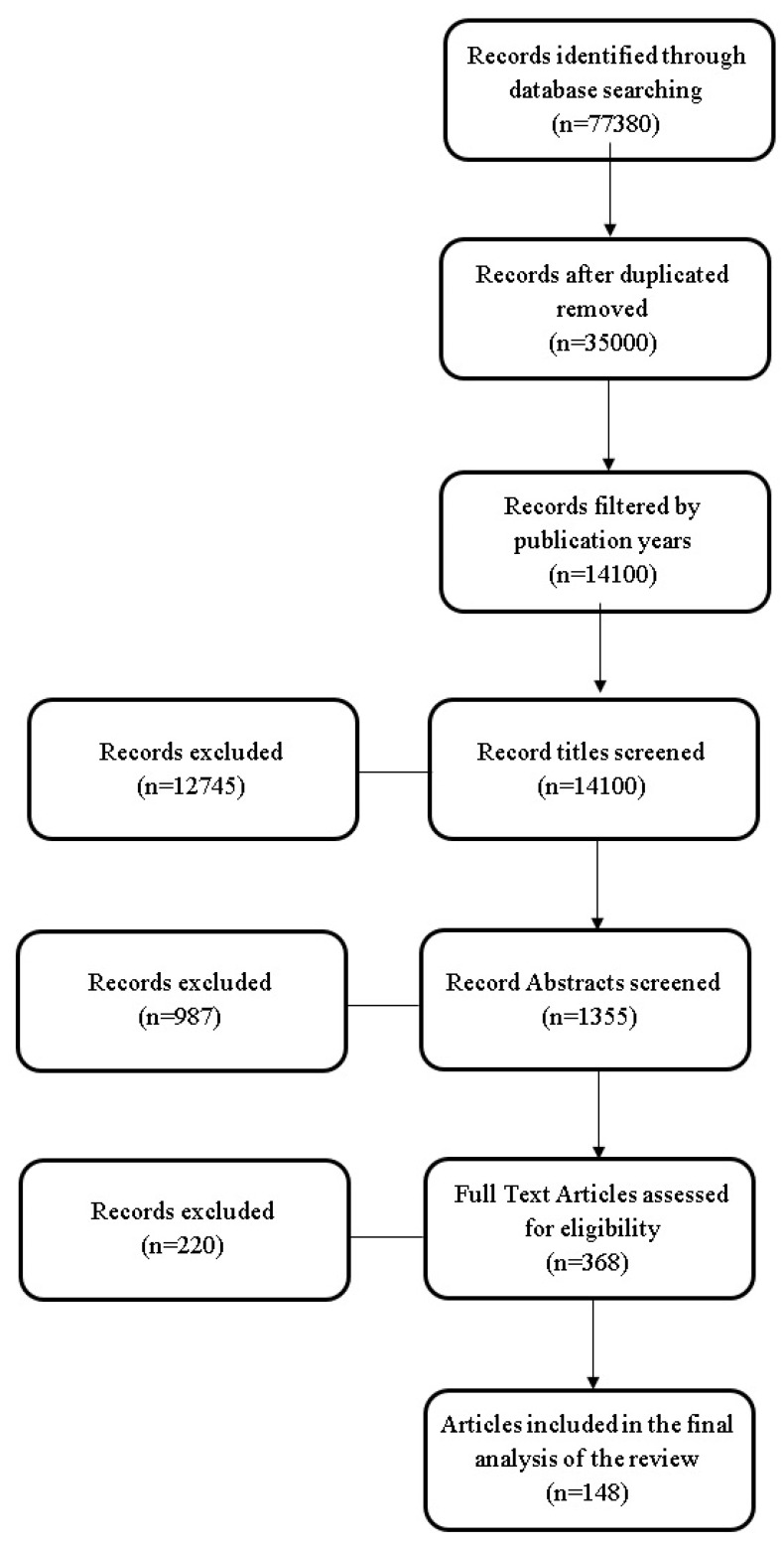
The review flowchart.

This systematic review included 148 studies, identified through systematic database searches using “gastrointestinal manifestations” and “cystic fibrosis” on PubMed and Google Academic. After title screening, 12,745 articles were excluded due to title–research objective mismatch. Abstract examination excluded more articles based on relevance, publication date, accessibility, or pertinence. Thorough text analysis led to the exclusion of 218 articles due to relevance, methodological incongruity, scope misalignment, quality issues, or language barriers. The accompanying flowchart (Figure 1) illustrates the sequential progression of information through the review process, depicting the tally of records ascertained, incorporated, and eliminated.

### 2.2. Study Selection

The article selection and curation for our review adhered to strict criteria, including alignment with central research questions on G-I manifestations of CF in children, considering diagnosis and management. The criteria involved research objectives, publication year, scientific categorization, and presentation quality. Searches were precisely structured with keywords and Boolean operators.

After data extraction, selected articles underwent meticulous categorization and synthesis within a structured framework, forming the review’s foundation. Qualitative analysis scrutinized scholarly adherence, clarity, brevity, citation frequency, sample size, data relevance, results articulation, and conclusions formulation. These facets were amalgamated into the final narrative synthesis.

## 3. Results

### 3.1. Gastrointestinal Manifestations in CF—General Data

CF exerts its influence on the G-I tract from the uterine and neonatal stages, persisting throughout an individual’s life [1,9]. G-I symptoms, observed in approximately 85% of cases, tend to be more prevalent in patients with severe disease or genotypes associated with moderate or severe abdominal involvement, potentially contributing to heightened morbidity and mortality among CF patients [10]. This phenomenon arises due to inadequate pancreatic enzyme release into the intestine, resulting in impaired food digestion. The multifactorial etiology of these manifestations involves CFTR dysfunction, a high-fat CF diet, and antibiotic use. Abdominal symptoms serve as a distinctive feature of multiorgan CF involvement, with patients undergoing intravenous antibiotic therapy often experiencing more pronounced G-I symptoms [11].

**Figure 2:**
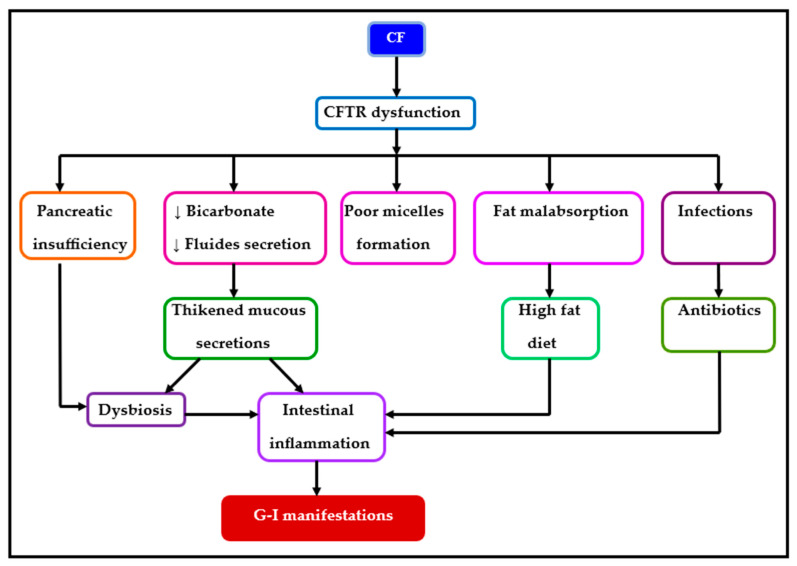
Etiology of G-I manifestations in CF (Legend: ↓ = decreased).

G-I impairment in CF is attributed to altered intestinal secretion, the absence of pancreatic fluids containing enzymes, dysbiosis, and intestinal inflammation. In the digestive tract, CFTR functionality is crucial for water and ion homeostasis, with a strong expression of the CFTR gene in the stomach and particularly in the intestinal tract. CFTR dysfunction adversely affects smooth-muscle contractility, leading to consequences such as pancreatic insufficiency, reduced bicarbonate, and fluid secretion, resulting in the formation of viscous secretions and fat malabsorption [12]. The CF gut operates within a deleterious cycle involving impaired luminal flux due to the viscous mucus layer, epithelial inflammation, infection, and/or dysbiosis [13]. The G-I damage observed in CF is attributed to the alteration of intestinal secretion, absence of pancreatic fluids containing enzymes, dysbiosis, and intestinal inflammation, as illustrated in Figure 2 [1,8,14,15].

The dehydration of secretions leads to intraductal blockage, inflammation, fibrosis, and potential organic destruction in the presence of digestive enzymes [16,17,18,19]. Several studies report a reduction in β-cell area, ranging from 11% to 52%, while others indicate an unchanged number of pancreatic cells. In the proximal intestine, increased bicarbonate secretion fails to adequately neutralize gastric acid, contributing to imbalances. In pancreatic CF patients, low bicarbonate levels and secretion volume are observed, but the flow remains sufficient to support necessary enzyme secretion for digestion [14].

G-I symptoms in CF patients exhibit an inverse correlation with dietary fiber content. Studies on mice suggest that fiber reduces the paracellular permeability induced by oleic acid or reserpine, and elevated enzyme doses lead to intestinal eosinophilia and necrosis [20]. Emerging evidence highlights the significant role of intestinal inflammation in the manifestation of G-I symptoms in CF, with multifactorial causes contributing to inflammation.

Within the small intestine lumen of individuals with CF, elevated levels of inflammatory markers and morphological abnormalities, such as edema, erythema, ulceration, and destruction, are observed. Studies on mice indicate that intestinal inflammation correlates with reduced activity of ligand-dependent type-II nuclear receptors, impacting the metabolism and transport of fatty acids, sterols, bile acids, and xenobiotic acids. The modification of the intestinal microbial environment contributes to inflammation and the subsequent deterioration of the protective bacterial barrier in CF patients [21,22]. Inflammation further enhances *Escherichia coli* colonization of the intestinal mucosa. Conversely, *E. coli* can contribute to inflammation, impairing metabolism and lipid absorption, leading to malnutrition and symptom exacerbation [1]. Antibiotic use is recognized as an iatrogenic factor in CF associated with gut inflammation. Knoop et al. (2016) observed in a mouse study that oral antibiotic administration leads to increased inflammatory cytokines (IL-17, IFN-γ, and chemokine C-X-C motif ligand 1) alongside alterations in gut microbial composition [23]. Prolonged exposure to antibiotics in CF patients exacerbates the alteration of gut microbial composition [24,25,26,27]. During antibiotic treatments in CF, butyrate-producing strains, such as *Anaerostipes*, *Butyricicoccus*, and *Ruminococcus*, are diminished [21]. Regarding cumulative intravenous antibiotic use over one year, Bruzzese et al. (2014) reported a negative correlation between the number of intravenous antibiotic courses and gut microbiota diversity. The highest exposure to intravenous antibiotics was associated with the lowest proportions of *Bacteroidetes* and the highest proportions of *Firmicutes* [28]. 

In the short term, intestinal inflammation demonstrates an impact on nutritional status, as demonstrated by the correlation between calprotectin levels and Z-scores for weight and waistline. Over the long term, inflammation influences morbidity and mortality, particularly elevating the risk of colon cancer [29]. Chronic inflammation and perturbation of the gut microbiome are prevalent among individuals with CF. Research indicates heightened levels of inflammatory proteins in the intestine, increased fatty acids in stools, and endoscopic findings, revealing villous atrophy, edema, erythema, and mucosal ulceration. These histological manifestations are associated with elevated calprotectin levels in stools [13]. Nonetheless, although increased calprotectin levels in stools do not precisely predict intestinal inflammation in CF, their elevated values across both the pancreatic sufficiency and pancreatic insufficiency groups studied substantiate the concept of “enteropathy” in CF, irrespective of pancreatic status [30].

### 3.2. Gut Dysbiosis 

In CF, gut dysbiosis manifests shortly after birth, impacting the intestine [14]. Dysbiosis arises from disruptions in microbiome cell density and diversity, antibiotic effects, and alterations in the luminal environment and small intestine physiology, as well as mucus and mucin accumulation. These factors influence both intestinal and extraintestinal manifestations [1,9,13,14]. Contributing factors to dysbiosis encompass hydro-electrolyte imbalances, intestinal exocrine dysfunction, slowed G-I transit time, impaired intestinal immunity, ingestion of infected mucus, severity of CFTR gene mutations, and a hypercaloric diet [29,31].

Alterations in the microbiome are associated with intestinal inflammation in CF, characterized by a deficit in species like *Bacteroides*, *Bifidobacterium adolescentis*, *Faecalibacterium prausnitzii*, and *Eubacterium* spp., accompanied by an increase in opportunistic bacteria such as *E. coli* and *Eubacterium biforme* [29,31,32,33]. Pediatric CF samples notably exhibited a higher *E. coli* prevalence compared to non-CF samples. Elevated *E. coli* concentrations in the colon correlate with inflammatory processes and carcinogenesis initiation in inflammatory bowel disease, suggesting a link between dysbiosis, particularly the overrepresentation of *E. coli*, and the development of chronic G-I pathology [34].

Intestinal motility and soluble mucins regulate bacterial load in the proximal intestine, while mucus adhesion and lubrication influence bacterial binding to complex oligosaccharides on mucin molecules. Abnormal colonization results from sticky mucus and slowed motility, leading to increased proinflammatory species (*E. coli*, *Enterobacteriacee*, *Bacteroides fragilis*, *Mycobacterium*, *Proteobacterii*, *Streptococcus*, and *Veillonella*) and reduced beneficial bacteria (bifidobacteria, *Akkermansia*, *Eggerthella*, and *Anostipes*). Specific microbial profiles are associated with distinct CF characteristics, such as elevated *E. coli* and *E. biforme* levels in F508del mutation carriers, increased *F. prausnitzii*, bifidobacteria, and *E. limosum* in those with moderate disease, and an altered *Firmicutes–Bacteroidetes* ratio in those with liver damage. An abundance of *Staphylococcus* and *Faecalibacterium* negatively correlates with body mass index (BMI) in CF patients, and increased Alistipes levels impact glucose homeostasis. Some CF patients harbor *Clostridioides difficile* in stool, often remaining asymptomatic or having nontoxigenic strains [9,13]. Notably, CF-associated loss of *Oxalobacter formigenes*, a microorganism metabolizing oxalates, increases the risk of hyperoxaluria and kidney stone formation [1,14]. Table 1 summarizes the findings from various studies regarding alterations in gut microbiota observed in individuals with CF.

Several mechanisms intervene in the production of CF dysbiosis:

- Mechanisms related to CFTR:
  - Thick mucus due to chloride channel dysfunction;
  - Deficient bicarbonate secretion that alters intestinal pH;
  - Malabsorption due to pancreatic insufficiency;
  - Intestinal dysmotility with prolonged transit time;
  - Altered immune mechanisms in the mucosa;
  - Increased inflammation;
  - Damage to the intestinal barrier.
- Acquired factors:
  - Frequent use of antibiotics for recurrent respiratory infections;
  - Hypercaloric, hyperlipidic diet;
  - Use of other drugs (inhibitors of acid secretion, opioids, anticholinergics, immunosuppressants) [9].

Dysbiosis is associated with fat malabsorption, as evidenced by the heightened prevalence of *E. coli* in individuals with CF, indicating a positive correlation with intestinal inflammation and disturbances in lipid metabolism and absorption, further exacerbating malnutrition [41]. Identifying dysbiosis in infants and young children may offer an opportunity for intervention, enabling therapeutic modulation of their gut microbiota. Early onset of lung disease and intestinal dysbiosis mutually influence each other [9]. The clinical symptomatology observed in dysbiosis resembles that of bacterial overpopulation syndrome.

#### 3.2.1. Diagnosis of Gut Dysbiosis

The tests that are useful for identifying gut dysbiosis are:

- Stool test that measures the amount of good and bad bacteria in the stool;
- Organic acid test that measures the number of organic acids in the urine, bacteria producing organic acids as by-products of metabolism;
- Hydrogen breath test that measures the amount of hydrogen exhaled after drinking a sugar solution and breathing into a test tube;
- DNA analysis tools for gut microbiota, used to identify and quantify disease severity [42].

New research methods on gut microbiota in CF patients include a multiomics approach, involving metagenomics, metatranscriptomics, metaproteomics, and metabolomics [43]. 

#### 3.2.2. Treatment of Gut Dysbiosis 

Methods of modulation of dysbiosis in CF are recommended:

- Change in the composition of macronutrients;
- Micronutrient supplementation;
- Administration of prebiotics, probiotics, symbiotics, postbiotics, and flavonoids [9].

Human-milk oligosaccharides contribute to elevated *Bifidobacterium* levels in the infant’s gut, influencing acetate production and acting as a preventive measure against *E. coli* infection. Feeding infants with human milk enhances gut microbiome diversity and diminishes respiratory tract colonization [9]. In the context of CF, probiotics have demonstrated positive effects on gut motility, inhibition of bacterial colonization, enhancement of intestinal barrier function, improvement in metabolic processes, and modulation of immunity [44]. The administration of probiotics (specifically, *Lactobacillus rhamnosus* GG at one capsule/day, and *Lactobacillus reuteri* at five drops/day, continuously) proves beneficial in reducing markers of intestinal inflammation, as indicated by decreased fecal calprotectin and rectal nitric oxide levels, although such interventions are not yet part of routine prescription practices [9]. A recent study by Asensio-Grau et al. (2023) emphasized that supplementation with *Lacticaseibacillus rhamnosus*, *Limosilactobacillus reuteri*, and *Lactiplantibacillus plantarum* induced modifications in the colonic microbiota, reducing *Proteobacteria* and *Bacteroidota* levels while increasing *Firmicutes* abundance [45]. 

Nutritional factors impact the fecal microbiome, with nutritional supplements and high-calorie, high-fat, processed foods influencing the gut microbiota. However, it is essential to note that gut dysbiosis can also impact nutrient absorption. Treatment for *C. difficile* infection involves Metronidazole or Vancomycin administered for 10–14 days [13,46]. Furthermore, Ivacaftor (IVA) treatment has been associated with an elevation in *Akkermansia* species, known for mucosal protection, and this increase is negatively correlated with stool markers of inflammation [9].

### 3.3. Small Intestinal Bacterial Overgrowth (SIBO)

Impairment to peristalsis, antibacterial proteins, gastric acid, intestinal fluids, and the ileocaecal valve leads to bacterial overgrowth in the small intestine, representing a form of intestinal dysbiosis. This condition can advance to abdominal distension, flatulence, steatorrhea, weight loss, diarrhea, and macrocytic anemia. Concurrently, mucus accumulation, mucosal immune dysfunction, water and electrolyte imbalances, and malabsorption contribute to alterations in the nutrient pool within the G-I lumen [1]. The observed weight loss can be attributed to bacterial competition for ingested nutrients, the presence of inflammation, and the bacterial capacity to deconjugate bile acids, diminishing their efficacy in emulsifying fats [14].

SIBO is prevalent in approximately 30–40% of CF patients. It arises from the accumulation of thick mucus and the compromise of normal bacterial defenses, manifesting as the presence of over 10 colony-forming units/mL in the small intestine. The heightened bacterial load stimulates mucus secretion, perpetuating a detrimental cycle between mucus plaque formation and dysbiosis. SIBO may be linked to intestinal dysmotility and the malabsorption of essential nutrients, such as iron, vitamin D, vitamin B12, bile acids, and folates [13,47].

#### 3.3.1. Diagnosis

SIBO encompasses a spectrum ranging from nonspecific abdominal manifestations (abdominal cramping or pain, steatorrhea, anemia, weight loss, abdominal distension, flatulence, fatigue, and poor concentration) to more severe outcomes like malnutrition and malabsorption [48,49]. 

The “gold standard” diagnostic method for SIBO involves an aspirate culture with a bacterial count of ≥10^3^ CFU/mL. However, due to its invasive nature, this method is not recommended for use in pediatric populations [49,50]. An alternative, noninvasive diagnostic approach employs the hydrogen breath test, with a positive outcome indicative of SIBO when hydrogen levels exceed 12 parts per million (ppm) [49,51]. In a study by Gabel et al. (2022), 73.7% of CF patients demonstrated a positive breath test, suggesting the presence of SIBO [52]. Another diagnostic alternative to the breath test involves the utilization of orally ingested capsule technology, which measures in vivo hydrogen and carbon levels following carbohydrate ingestion [49]. 

#### 3.3.2. Treatment

Due to diagnostic limitations, empirical treatment is commonly employed for SIBO, with the resolution of clinical symptoms serving as confirmation for this syndrome.

Typically, SIBO treatment involves the administration of antibiotics, such as Metronidazole at a dosage of 20 mg/kg body weight/day, Rifaximin at 200 mg twice daily for children aged 3–11 years, and 550 mg twice daily for those over 12 years, Trimethoprim/Sulfamethoxazole at 12 mg/kg body weight/day, and Amoxicillin-clavulanate. A rotational strategy, alternating antibiotics every two weeks, is implemented to mitigate the risk of bacterial resistance development [13,47]. It is crucial to acknowledge that antibiotic usage impacts the normal commensal bacterial flora and may contribute to selective bacterial resistance. Additionally, antibiotic use raises the potential for *Clostridioides difficile* infection, triggering secretory diarrhea through the toxin-induced activation of CFTR-dependent chloride secretion [1].

Studies investigating the efficacy of probiotics in the context of SIBO have yielded conflicting outcomes. According to Dorsey et al. (2017), probiotics may serve as a viable treatment for SIBO, contributing to alterations in the fecal microbiome and a reduction in markers of inflammation [13]. Probiotic use in SIBO has been associated with a decrease in hydrogen (H2) levels and an elevation in decontamination rates. Zhong et al. (2017) suggest that probiotics are effective in diminishing the bacterial burden in SIBO, while the study by Husebye et al. (2001), conducted on rats, indicates that probiotics administered in SIBO exhibit prokinetic effects [53,54]. Dual therapy involving probiotics, such as *L. casei*, in conjunction with antibiotics, results in superior symptom improvement compared to the administration of antibiotics alone, as reported by Rosania et al. (2013), a finding corroborated by Khalighi et al. (2014), who assert that *Bacillus coagulan* probiotics administered during antibiotic therapy for SIBO can be beneficial in preventing complications [55,56]. It is noted that optimal outcomes are achieved by combining probiotics with rifaximin or minocycline [57]. However, the study by Rao et al. (2018) observes opposing effects, accentuating digestive symptoms following probiotic administration [58]. Aslan et al. (2023), in a study on rats treated with probiotics in conjunction with various essential oils (coconut, peppermint, lemon, and patchouli), demonstrated reduced proinflammatory cytokine levels (IL-1β, IL-6, and TNFα), histological improvement, and decreased inflammation [59]. The study by Kumar et al. (2018) concludes that the administration of *Bifidobacterium infantis* 35624 leads to an increase in methane levels in the breath test, while Zhong et al. (2017) determine that probiotic administration does not improve abdominal pain or stool frequency in SIBO patients [54,60]. Similarly, the results of the study by Khalighi et al. (2014), cited by Chen et al. (2014), indicate that probiotics do not significantly enhance pain, bloating, and diarrhea, with improvements in nausea, vomiting, and constipation similar to those observed in the control group. This study also reveals that the administration of symbiotics leads to the resolution of G-I symptoms in SIBO [61]. The study by Mitten et al. (2018) concludes that probiotic administration increases the risk of associating predominantly methanogenic SIBO forms with constipation [62]. 

The use of laxatives in the eradication of SIBO is posited to contribute to the normalization of intestinal transit [63]. In a study by Gabel et al. (2022), the administration of Lumacaftor/Ivacaftor (LUM/IVA), CFTR modulators, for one month did not result in significant changes in the breath test, with 65.8% of the patients still exhibiting a positive test [52]. 

Nutritional recommendations entail a reduction in fermented foods and the avoidance of fiber-rich products, polyols, sweeteners, and prebiotics [51]. The low fermentable oligosaccharides, disaccharides, monosaccharides, and polyols (FOODMAP) diet is considered optimal for individuals with SIBO, promoting the proliferation of less pathogenic bacteria [64]. Additionally, vegetarian and vegan diets appear to be more effective in managing SIBO [65]. 

### 3.4. Motility Disorders

The modulation of gut motility is orchestrated by the interplay among the gut luminal environment, immune system, enteric nervous system, and central nervous system. In individuals with CF, gastric motility may be impacted by a reduction in overall gastric secretion, leading to heightened viscosity and electrolyte concentration. Gut motility disorders observed in CF contribute to:

- Development of SIBO;
- Reducing the solubility of bile salts;
- Deconjugation of bile acids;
- Reducing intestinal absorption of bile acids;
- Excessive loss of bile acids in stools [66].

A correlation between dysbiosis and dysmotility exists, though it remains unclear whether dysbiosis serves as the cause or consequence [12]. Lewindon et al. (1998), as cited by Avelar-Rodriguez et al. (2019), observed a prolonged orocecal transit time in individuals with CF, potentially elevating the risk of SIBO [57]. SIBO, characterized by the presence of species such as *E. coli*, can contribute to intestinal dysmotility, leading to a deceleration of intestinal movements.

Within the initial 30 min following gastric evacuation, the small intestine exhibits abnormal acidity, facilitating the formation of mixed micelles with bile salts and lipid digestion products. This temporal window is crucial for the dissolution of pancreatic enzymes, influencing their efficacy. The administration of enzymes in the form of enteric-coated microcapsules ensures their passage through the acidic stomach lumen unaffected, subsequently dissolving in the less-acidic pH of the duodenum [1].

#### 3.4.1. Diagnosis

In human investigations, Hedsund et al. (2012) utilized a radio-opaque marker and reported a significant prolongation in the orocecal transit time in individuals with CF compared to their healthy counterparts [67]. More recently, Ng et al. (2021) employed innovative magnetic resonance imaging (MRI) techniques, demonstrating prolonged orocecal transit times in CF patients, concurrently associated with an augmented colon volume [15]. Gastric emptying scans are considered the optimal diagnostic method for evaluating gastric emptying. Other contemporary methods facilitating the diagnosis of delayed gastric emptying include wireless luminal imaging, transit-time recording using the Pillcam or the Smart pill, and the 3D-Transit system [13]. Utilizing endoscopic capsules, Malagelada et al. (2020) observed a notable reduction in intestinal contractility, accompanied by increased retention of intraluminal content in individuals with CF compared to their healthy counterparts [68].

#### 3.4.2. Treatment of Dysmotility 

Given the interplay among the immune system, G-I secretions, microbiota, and fermentation byproducts in modulating gut motility, the utilization of probiotics appears promising for mitigating G-I dysmotility. Probiotics, such as *Lactobacillus rhamnosus* GG, exhibit potential in modulating mucosal and systemic immune barriers, consequently normalizing inflammation-related dysmotility, as evidenced by studies conducted by Guarino et al. (2008) and Isolauri et al. (2001) [69,70]. However, there is insufficient data regarding the role of prebiotics in G-I dysmotility. Dietary fiber consumption is reported to have a positive impact on stool consistency and frequency, as confirmed by other authors [71]. DeLisle et al. (2013) and Quigley et al. (2013) propose that probiotics positively influence the G-I tract by ameliorating visceral hypersensitivity, dysmotility, and permeability, albeit with limited impact on the intestinal microflora [14,72]. Studies conducted on mice have demonstrated that a novel molecule called “Oligo G” can reduce mucus levels and enhance intestinal transit [73]. 

### 3.5. Malabsorption Syndrome

Malabsorption in CF manifests as a severe and early-onset condition, exacerbated by the frequently observed delayed gastric evacuation in CF patients [74]. Maldigestion and lipid malabsorption are intensified by hyperacidity in the duodenum, typical in CF, along with pancreatic insufficiency-induced lipase deficiency and reduced bile acid resorption in the ileum [1]. Proteins and carbohydrates absorption and digestion seem to be less affected, with maltase and sucrase activities appearing unaltered, while lactase activity is notably lower or suppressed [75].

The multifactorial etiology involves dysfunction of the endocrine pancreas and liver, impaired bile acid metabolism, and intestinal resorption processes [76]. Nonetheless, pancreatic enzyme deficiency stands out as the primary cause of malabsorption. Contributing factors encompass bicarbonate deficiency, abnormalities in bile salt, disruptions in mucosal transport, various motility issues, abnormal intestinal mucus, structural anatomical changes, dysfunction in enteric circular muscles, defects in mucosal mechanisms leading to the abnormal release of lipoproteins into the bloodstream, intestinal bacterial overload, and inflammatory processes [74,75,77,78].

CF malabsorption manifests through impaired pancreatic enzyme release and intestinal damage, hindering nutrient absorption. Severe pancreatic damage in exocrine pancreatic insufficiency results in reliance on lingual and gastric lipase for lipolysis [79]. The factors contributing to persistent fat malabsorption in CF include:

- Intestinal pH: CFTR dysfunction reduces bicarbonate secretion, leading to a lower pH. Improper mucin expansion due to disrupted bicarbonate secretion impedes lipid translocation, necessitating an increased pH for optimal fat absorption. Acid-suppressing treatments can enhance fat absorption but may induce SIBO and alter bile salt metabolism [13,79]. Cases of ongoing fat malabsorption despite enzyme-replacement treatment and low intestinal pH have been reported [80,81]. Stool pH correlates with fat absorption, possibly explaining enzyme-replacement ineffectiveness [81];
- Intraluminal bile salts: CF patients exhibit increased bile salt loss in stools due to mucosal changes (thickened mucus, SIBO). An elevated glycine–taurine ratio and reduced bile solubilization capacity affect fat absorption and decrease the bile salt pool. Even CF patients without liver damage experience bile acid loss in stools, contributing to malabsorption [14,79,82];
- Abnormalities of the gut mucosa: thick and adherent mucus, bacterial overgrowth, ileal hypertrophy, villous atrophy, increased permeability, and chronic inflammation contribute to fat malabsorption [79];
- Reduced orocecal intestinal transit time: fat absorption is influenced by the duration that fats are in contact with the absorption surface [79];
- Deficiency of essential fatty acids: low linoleic and docosahexaenoic acid levels, along with elevated arachidonic acid, can impede fat absorption, contributing to inflammation, mucus secretion, and smooth muscle relaxation [79].

#### 3.5.1. Diagnosis

Clinical indicators of malabsorption in CF include inadequate weight and height gain in pediatric patients, while low BMI in adults may suggest malabsorption. Confirmatory laboratory assessments include stool fat evaluation, optical microscopy for fat droplets, acid steatocrit, and the 13C-mixed triglyceride breath test [83].

Traditional verification of fat malabsorption involves measuring fecal fat excretion over at least three days, assessing concurrent food intake, and quantifying fat intake and production for a percentage fat absorption calculation. An alternative, validated, semiquantitative method modifies the steatotrit technique through fecal homogenate acidification, requiring stool-specimen centrifugation [83].

The 13C-mixed triglyceride breath test provides a safe and repeatable, albeit costly and less accessible, means of assessing fat digestion [84,85]. Annual evaluations, combining clinical and laboratory methods, are recommended for all CF patients [86].

Histologically, most CF patients exhibit a normal brush-border appearance, but some may present ileal hypertrophy or partial villous atrophy in the small intestine due to acid aggression, chronic inflammation, or denutrition [87].

#### 3.5.2. Treatment 

Treatment of malabsorption syndrome has the following objectives:

- Control of symptoms;
- Correction of malabsorption;
- Obtaining a nutritional status and growth as close to normal as possible [77].

Effective enzyme-replacement therapy may enhance lipid absorption [74,88]. Despite treatment, lipid malabsorption persists in some cases, with absorption coefficients below 85–90% of dietary lipid intake [79]. The cause of persistent malabsorption extends beyond pancreatic insufficiency, involving abnormal interenterocytic events impacting plasma lipid transport [78]. Fat-balance measurement is the “gold standard” for enzyme-replacement therapy monitoring [82].

Supplementing with essential fatty acids (70 mg/kg body weight of docosahexaenoic acid for 6 weeks) and regular use of omega-3 fatty acids did not improve fat absorption and nutritional status [79]. Due to increased mucus viscosity in the CF intestinal epithelium, N-acetylcysteine administration, breaking disulfide bonds, may be beneficial [76].

### 3.6. Meconium Ileus

Observed in 10–20% of CF patients, it represents the earliest G-I manifestation [12,47,89,90]. Its pathophysiology, though not fully elucidated, is hypothesized to stem from CFTR dysfunction in the enteric nervous system, particularly affecting the ileal response to abnormal intraluminal content [12]. Common mutations include F508del, G542X, W1282X, R553X, and G551D [35], with approximately 67% survival [90].

#### 3.6.1. Diagnostic

Manifestations emerge within the first 2 days, featuring signs of intestinal obstruction (abdominal distension, bile vomiting, delayed mucus elimination). Examination may reveal an abdominal mass in the right iliac fossa or supra pelvic region. Complicated cases may exhibit perforations, volvulus, or atresia [90,91]. Most cases involve pancreatic insufficiency [92]. Abdominal radiography shows dilated loops with hydro-aerial levels and an intra-abdominal mass, occasionally indicating meconial peritonitis [93].

#### 3.6.2. Treatment

Treatment involves intravenous rehydration and barium enema with Gastrografin^®^ or N-Acetylcysteine. Surgical intervention becomes necessary in cases with complications [90,94].

### 3.7. Rectal Prolapse

Rectal prolapse affects 3.5% of CF patients, with 3.6% of those experiencing prolapse having CF. Causes include constipation and heightened intra-abdominal pressure from coughing. Unexplained prolapse necessitates iontophoresis [95].

#### Treatment

Manual reduction, with the patient in the genupectoral position, constitutes the primary treatment. For recurrent cases, options include sclerotherapy or surgical intervention [93].

### 3.8. Intussusception

Intussusception is 10–20 times more prevalent in CF patients aged 9–12 years. Twenty-five percent of cases manifest as ileo-colonic forms. CFTR dysfunction, altered luminal environment, mucus clearance, abnormal bacterial colonization, dysbiosis, and appendiceal mucocele contribute to its etiology [1].

#### 3.8.1. Diagnosis

Intussusception presents with severe cramp-like pain, vomiting, abdominal distension, dehydration, and lethargy. Palpation reveals a lower right quadrant mass, requiring differentiation from distal intestinal obstruction syndrome. Mechanically described as “telescoping”, it commonly involves the ileum and cecum, with the mesentery’s potential incorporation elevating the risk of ischemia and necrosis [96]. An unusual manifestation results from CF-induced constipation, demanding a thorough diagnostic evaluation to exclude other CF-associated G-I conditions [97].

#### 3.8.2. Treatment

Management includes nasogastric tube placement for distension, intravenous electrolyte correction, nonopioid analgesics, and surgical intervention, if necessary [46]. Adewale et al. (2019) advocate conservative measures before surgery, emphasizing pancreatic enzyme use to prevent constipation and subsequent fecaloma formation, a potential trigger for intussusception [98].

### 3.9. Volvulus

Volvulus occurs in 15% of CF patients, causing 3–4 times proximal intestinal dilation. Meconium ileus complicated with volvulus poses a life-threatening scenario [99].

#### 3.9.1. Diagnosis

Often diagnosed prenatally through ultrasound, it manifests as a hyperechoic intestine with ascites and abdominal distension. Decreased fetal movements and incidents like volvulus necessitate CF testing at birth [100].

#### 3.9.2. Treatment

Intestinal volvulus is an urgent, life-threatening condition, particularly in confirmed or suspected CF cases. Emergency cesarean section and surgical resolution, involving intestinal resection, are imperative to mitigate absorption-function impairment [99].

### 3.10. Gastric and Duodenal Complications

*Helicobacter pylori* infection, inadequate gastric acidity neutralization, and prolonged antibiotic use contribute to gastroduodenal issues in CF [101,102]. *H. pylori* prevalence and cross reactivity with anti-*Pseudomonas* antibodies were explored in CF patients [103].

#### 3.10.1. Diagnosis

Despite treatment, severe G-I symptoms persist in some patients, necessitating endoscopy with biopsy. Noninvasive *H. pylori* testing (fecal antigen test or urea breath test) may be warranted for those with dyspepsia or suspected peptic ulcer disease [104]. Gastroparesis prevalence rises with age in CF, with scintigraphy recommended for diagnosis [105].

#### 3.10.2. Treatment

Gastroparesis treatment includes prokinetic agents, macrolides, gastrostomy, and gastro-jejunostomy tubes [78]. Inflammation on biopsy warrants immunomodulatory agents [106]. IVA and LUM/IVA modulators improve weight gain in CF, likely linked to reduced stool calprotectin and enhanced dietary-fat absorption [107,108]. IVA normalizes intestinal pH, decreases calprotectin, and increases *Akkermansia* abundance, resolving histopathological changes [40,47,107,109,110]. Modulator-induced intestinal inflammation mitigation may positively influence CF patient growth and nutrition [110].

### 3.11. Gastro-Esophageal Reflux (GER)

Less-specific GER symptoms are common in CF, particularly in youth [6,12]. CF patients with GERD experience worsened lung disease progression due to refluxed contents containing acid, enzymes, and bacteria [111]. Lower gastro-esophageal sphincter pressure reduction, elevated intra-abdominal pressure from chronic cough, and increased gastro-esophageal pressure gradient contribute to GER, which is intensified by higher negative intrathoracic pressure in CF [112,113].

This pressure difference may increase the gastroesophageal pressure gradient, leading to GER. Dysfunctional esophageal sphincter pressure contributes to increased proximal reflux, causing prolonged exposure to acidic contents, elevating GERD symptoms, and exacerbating lung issues [114,115].

#### 3.11.1. Diagnosis

GER may cause pain and complications, requiring exploration of pepsin as an aspiration biomarker [115]. Esophageal manometry reveals coughing following reflux episodes [91]. For GER patients with Barrett’s esophagus, endoscopy screening is recommended [111].

#### 3.11.2. Treatment

No CF-specific GER treatment guideline exists; chronic PPI use correlates with a potential exacerbation risk [111,116,117]. PPIs improve esophageal acid exposure and the DeMeester Score [118]. Surgical intervention (Nissen fundoplication) is required for cases with unfavorable outcomes [91].

Zeybel et al. (2017) observed reduced GER symptoms during ivacaftor (IVA) administration over 52 weeks, with alkalization of the intestinal pH and improved enzyme functionality [118]. Ongoing studies assess Elexacaftor, Tezacaftor, and Ivacaftor (ETI) modulators, revealing improvements in GERD symptoms after 3 and 6 months of ETI administration [119,120].

### 3.12. Eosinophilic Esophagitis (EoE)

In CF, EoE, a chronic inflammatory disorder characterized by esophageal eosinophilic infiltration (≥15 eosinophils/field), presents with symptoms such as dysphagia, chest pain, burning sensation, abdominal pain, and growth difficulties [14,91,121]. The increased EoE prevalence in CF may result from factors like antibiotic exposure altering the G-I microbiome, higher GERD rates in CF patients, and associated PPI use, as well as an elevated prevalence of atopic manifestations [9,22,46,122]. 

#### 3.12.1. Diagnosis

Diagnosis involves endoscopy and esophageal biopsy, with a crucial differential diagnosis from GER [14].

#### 3.12.2. Treatment

Consensus guidelines propose three EoE treatment lines:

- Proton-pump inhibitor (PPI) use (20–50% success rate);
- Corticosteroid therapy (Budesonide, Fluticasone), with efficacy in 50–90% of cases;
- Dietary interventions (elimination of food allergens) [9,46,123,124,125].

### 3.13. Distal Intestinal Obstruction Syndrome (DIOS)

DIOS, a CF complication, results from the accumulation of viscous feces and mucus, causing obstruction in the terminal ileum and cecum [112]. Characterized by a complete or incomplete obstruction, it affects 10–22% of CF patients, with a higher incidence in older individuals and a 77% recurrence risk [90,126]. Risk factors include severe genotype, dehydration, meconial ileus history, and post-transplant complications, notably after lung transplantation [90].

#### 3.13.1. Diagnosis

Acute onset includes periumbilical or lower right quadrant pain, distension, and bile vomiting. Differential diagnosis involves constipation, intussusception, inflammatory bowel disease, and fibrosing colonopathy [126].

#### 3.13.2. General Treatment

- Laxatives;
  ◦ Osmotic drugs (lactulose, Macrogol 3350, Diatrizoate);
  ◦ Stimulants (Senna, Sodium docusate, Sodium picosulfate).
- Mucolytics: N-acetylcysteine (oral, diluted);
- Other agents: prokinetics (macrolides, Metoclopramide), Lubiprostone (Amitiza);
- Surgical procedures: considered for refractory cases [90,127].

Incomplete Forms Treatment:

- Oral rehydration, osmotic laxatives (PEG), magnesium citrate, or Gastrografin;
- Prokinetics are suggested in pseudo-obstructions or postoperatively [126,128].

Complete Forms Treatment:

- PEG in nonvomiting patients;
- Intestinal lavage;
- Rehydration (IV or Gastrografin enema);
- Cecum instillations (colonoscopy);
- Surgery (laparotomy, ileocecal resection) [126].

Prevention:

- Hydration;
- Laxatives;
- Adequate pancreatic enzymes;
- PEG 0.5–1 g/kg/day orally for 6–12 months [126].

CFTR Modulators:

Unclear impact; no specific studies conducted [129].

### 3.14. Constipation

Constipation, affecting over half of CF patients, is distinguished from DIOS, manifesting as gradual fecal impaction in the entire colon [90].

*ESPGHAN working group definition*:

1. Abdominal pain/distension;
  - Reduced bowel movement frequency;
  - Increased stool consistency.
2. Responds to laxatives [128].

Incidence is 1.5 times higher in pancreatic insufficiency. Contributing factors include meconium ileus history and fat malabsorption [111]. Colon barriers, vulnerable to compromise, may undergo destruction due to immune-cell infiltration and inflammation [1].

#### 3.14.1. Diagnosis

The presence of defined symptoms constitutes diagnosis.

#### 3.14.2. Treatment

There is no distinct CF approach; general-population treatments apply. Mineral oils are discouraged for those with lung disease [84]. The impact of CFTR modulators on constipation remains uncertain, with potential adverse events observed [130,131].

### 3.15. Colon Disease: Fibrosing Colonopathy

#### 3.15.1. Fibrosing Colonopathy

Fibrosing colonopathy, a severe, yet infrequent, condition, typically emerges in patients aged 2–7 years. While its pathogenesis remains elusive, prolonged high-dose pancreatic enzyme therapy is implicated, though occurrences in untreated patients exist. Predisposing factors include youth, colitis history, meconium ileus, DIOS, bowel surgery, antioxidant deficiency, and drug use (laxatives, corticosteroids, dornase alpha, and H2 receptor antagonists) [132]. Primarily affecting the ascending colon, the disease may progress throughout the colon, histologically characterized by submucosal fibrosis and elevated calprotectin correlating with colonic inflammation [14,112].

##### Diagnosis

Often presenting as treatment-resistant intestinal obstruction resembling DIOS, symptoms include abdominal pain, distention, vomiting, and constipation. Diagnosis consideration arises when standard DIOS interventions prove ineffective. Submucosal concentric rings, muscular mucosa hypertrophy, inflammatory cell infiltration, and collagen deposition typify the disease, suggesting recurrent ischemic events and mucosal reparative mechanisms [133]. Excessive pancreatic enzyme doses (>50,000 U lipase/kg) show a significant correlation with increased fibrous colonopathy risk [134].

##### Treatment

Prevention involves limiting lipase to 10,000 IU/kg/day. Nonresponsive cases necessitate treatment-adherence review, enzyme-preparation adjustments, timing changes, gastric acidity reduction, and exclusion of other G-I diseases. Surgical intervention, such as right hemicolectomy, is occasionally warranted [75,135,136].

#### 3.15.2. Crohn’s Disease 

Crohn’s disease prevalence is 12.5 times higher in individuals with CF than in the general population. Genetic and environmental factors and immunological interactions with intestinal microbiota contribute to this heightened occurrence [1,14].

##### Diagnosis

In CF, elevated markers of intestinal inflammation (IL-8, IL-1β, neutrophilic elastase, eosinophilic cationic protein, and plasma proteins) and increased calprotectin in stool are observed [21,28]. Although serum biomarkers for inflammatory bowel disease may yield false values, a rectal biopsy is recommended to enhance diagnostic accuracy [1].

##### Treatment

Immunosuppressant use, particularly Infliximab, is infrequent in CF patients. Relative contraindications include bronchiectasis and *P. aeruginosa* colonization [137].

### 3.16. Celiac Disease

Celiac disease exhibits a prevalence 2–3 times higher in individuals with CF than in the general population. This association, first described in 1969, involves complex interactions, including CFTR as a molecular target of gluten, contributing to CD pathogenesis [138,139].

The heightened prevalence varies globally, with increased occurrences noted in certain countries, emphasizing geographic distinctions [138,140,141,142]. CFTR inhibition by gluten disrupts ion balance, autophagy, and proteostasis, mirroring the stress response observed in FC. CFTR dysfunction, linked to increased intestinal permeability and inflammation, may predispose individuals to CD. Exocrine pancreatic insufficiency intensifies the antigenic load and antibody response [138,142].

Studies reveal that gluten-derived peptides inhibit CFTR, causing local stress responses contributing to CD immunopathology. The correlation involves factors like undigested gluten proteins, intestinal mucosa destruction, and immunological reactions against wheat gluten. Chronic inflammation in CF increases susceptibility to osteoporosis and colorectal cancer [140,143].

CFTR loss escalates reactive oxygen species and activates transglutaminase, leading to NF-KB activation and proinflammatory cytokine release. Malabsorption, high-calorie diets, and shorter duration of natural diet increase the risk of CD in CF patients [144].

#### 3.16.1. Diagnosis

CF and CD share malabsorption, clinical manifestations, and symptoms, posing challenges in differentiation. Liver damage, persistent anemia, and exocrine pancreatic insufficiency contribute to diagnostic complexities [115]. CF patients exhibit elevated antibodies, increased intestinal permeability, calprotectin levels, and microbiome alterations. Screening for CD is vital in CF patients with persistent symptoms, inappropriate growth, low BMD, and those requiring high pancreatic enzyme doses [14,99,138,139,140,145,146]. Pancreatic isoamylase activity distinguishes CF from CD in patients with steatorrhea [147].

#### 3.16.2. Treatment

CFTR modulators, like VX-770 (IVA), mitigate gliadin’s negative impact on CFTR function, reducing inflammation and inducing gluten tolerance in CD patients. CFTR modulators significantly enhance G-I symptoms and prognosis in both conditions [7].

### 3.17. Appendicular Disease

Appendicitis is uncommon in CF, presenting with atypical manifestations and a heightened risk of perforation and abscess formation. 

#### 3.17.1. Diagnosis

Diagnosing appendicitis in CF poses challenges, often characterized by an appendix diameter exceeding 6 mm due to luminal mucus. Delayed diagnosis, potentially confused with conditions like intussusception or DIOS, may lead to complications, such as appendicular abscess formation. Recognition of appendiceal mucocele involves identifying recurrent abdominal pain and palpable masses and confirmation through ultrasound examination [1,112,148].

#### 3.17.2. Treatment

Appendectomy, involving appendix resection and cecal tip removal, is effective in preventing recurrence of appendicitis or appendiceal mucocele. However, studies suggest that asymptomatic cases may not necessitate surgical intervention. Noninvasive approaches, such as mucin distension of the appendix, emerge as potential methods to safeguard CF patients against appendicular inflammation [148].

## 4. Discussions

The diminished levels of bicarbonate and the absence of bicarbonate-rich pancreatic fluids, essential for food digestion, contribute to the emergence of G-I manifestations in CF. These manifestations significantly impact the patient’s quality of life and long-term prognosis. While extensive research has been dedicated to pulmonary aspects, there is a notable scarcity of high-quality studies on G-I manifestations in CF. Therefore, further investigations are imperative to comprehensively assess the scope of G-I manifestations, the long-term risks associated with this pathology, and the potential impacts of emerging therapies specific to G-I manifestations.

Despite limited clinical studies on the efficacy of probiotics in managing CF patients, the presence of gut dysbiosis necessitates interventions targeting the gut microbiota. Probiotics such as *Lactobacillus reuteri* and *Lactobacillus rhamnosus* GG hold promise for conferring health benefits to CF patients. The heterogeneity in strains, doses, and treatment durations across existing studies underscores the need for large-scale investigations to elucidate the specific effects of these probiotics.

Given the early onset of G-I manifestations and the approval of CFTR modulators for older patients, it is crucial to explore their preventive effects on disease progression. Additionally, the discovery of novel CFTR modulators suitable for younger age groups warrants investigation. A worthwhile avenue for research involves examining the potential synergies between probiotics and CFTR modulators in alleviating CF G-I distress. Since existing studies primarily focus on the pulmonary effects of different CFTR modulators, future directions should encompass an exploration of their short-term and long-term impacts on G-I distress, including their role in preventing G-I cancer, along with considerations of optimal modulator-therapy timing.

## 5. Conclusions

Timely identification and intervention for gastrointestinal manifestations in cystic fibrosis hold significance not only in ameliorating symptoms but also in enhancing the nutritional status and overall survival of affected individuals. Given the intricate nature of G-I manifestations associated with CF, their effective management necessitates careful oversight and follow up, preferably administered through a collaborative effort involving a multidisciplinary healthcare team. This comprehensive approach is integral to optimizing care and elevating the quality of life for CF patients grappling with G-I complications.

Our contention underscores the imperative for further clinical investigations, aiming to establish a more robust and evidence-based framework for the management of G-I symptoms within the context of this chronic disease. Such scholarly endeavors are pivotal for advancing the understanding of the intricate interplay between CF and G-I manifestations, ultimately contributing to refined clinical strategies and improved outcomes for individuals facing these challenges.

## Figures and Tables

**Table 1:** Gut microbiota changes in CF.

Study	Author	Year	Increased Level	Decreased Level	Reference
Gut and respiratory microbiome in CF in infants	Madan et al.	2012	*E. coli*	*Staphylococcus* spp.	[35]
			*Parabacteroides* spp.	*Clostridium* spp.	
			*Veilonella* spp.		
Microbiota composition of the CF patients	Fouhy et al.	2017	*Enterobacteriaceae* spp.	*Faecalibacterium prausnitzii*	[36]
			*Clostridium* spp.	*Actinobacteria* spp.	
			*Enterococcus faecalis*	*Bacteroidetes* spp.	
			*Firmicutes* spp.	*Proteobacteria* spp.	
Gut microbiota signatures in CF	Vernochi et al.	2018	*Propionibacterium* spp.	*Eggerthella* spp.	[37]
			*Staphylococcus* spp.	*Eubacterium* spp.	
			*Clostridiaceae* spp.	*Ruminococcus* spp.	
				*Dorea* spp.	
				*Faecalibacterium prausnitzii*	
				*Lachnospiaceae* spp.	
Microbiota disturbances in children with CF	Enaud et al.	2019	*E.coli*	*Bacteroides* spp.	[29]
			*Eubacterium diforme*	*Bifidobacterium adolescentis*	
				*Faecalibacterium prausnitzii*	
Gut microbiota in children with CF	Coffey et al.	2019	*Fusobacteria* spp.	*Verrucomicrobia* spp.	[38]
			*Proteobacteria* spp.	*Firmicutes* spp.	
				*Ruminococcus* spp.	
				*Lachnospira* spp.	
Impact of CF on gut microbiota	Kristensen et al.	2020	*Enterococcus* spp.	*Bacteroides* spp.	[26]
			*Streptococcus* spp.	*Bifidobacterium* spp.	
			*E.coli*		
CF gut microbiome	van Dorst et al.	2022	*Enterococcus* spp. *Veilonella* spp. *Enterobacter* spp.	*Bacteroidetes* spp.	[39]
				*Ruminococcacceae* spp.	
				*Bifidobacterium* spp.	
				*Roseburia* spp.	
Intestinal microbiome in CF	Price et al.	2023	*Blautia* spp.	*Roseburia* spp.	[40]
